# The Synergistic Antibacterial Mechanism of Gentamicin-Loaded CaCO_*3*_ Nanoparticles

**DOI:** 10.3389/fchem.2017.00130

**Published:** 2018-01-23

**Authors:** Xiaohong Pan, Saili Chen, Dongzhe Li, Wenhua Rao, Yilin Zheng, Zhaoyuan Yang, Lan Li, Xiong Guan, Zhi Chen

**Affiliations:** ^1^State Key Laboratory of Ecological Pest Control for Fujian and Taiwan Crops, Key Lab of Biopesticide and Chemical Biology, Ministry of Education and Pharmaceutical Engineering, College of Plant Protection, Fujian Agriculture and Forestry University, Fuzhou, China; ^2^Fujian-Taiwan Joint Center for Ecological Control of Crop Pests, Fujian Agriculture and Forestry University, Fuzhou, China; ^3^College of Resources and Environmental Sciences, Fujian Agriculture and Forestry University, Fuzhou, China

**Keywords:** nano-CaCO_3_, gentamicin, antibacterial activity, drug loading, synergism

## Abstract

In the present study, we used CaCO_3_ nanoparticles (CCNPs) as carriers to assess the physicochemical characteristics and antibacterial effect of gentamicin sulfate (GS)-loaded CCNPs (CGPs). The results indicated that CCNPs had relatively regular chain-like structure, and the size of the crystallites was around 62.5 nm. FT-IR analysis indicated that the GS could effectively load onto CCNPs. Meanwhile, the dosage of CCNPs would affect the drug loading and entrapment efficiency of GS. CCNPs could prolong the release of GS, and the complete release of GS from CCNPs was extended up to 24 h. Additionally, CCNPs could obviously increase the antibacterial effect of GS. The zeta potential analysis and microscopic investigations indicated that the adsorbed CCNPs could increase the damage level of bacterial cell wall and enhance the permeability of cell membranes, leading to increased bacterial death.

## Introduction

**Figure 1:**
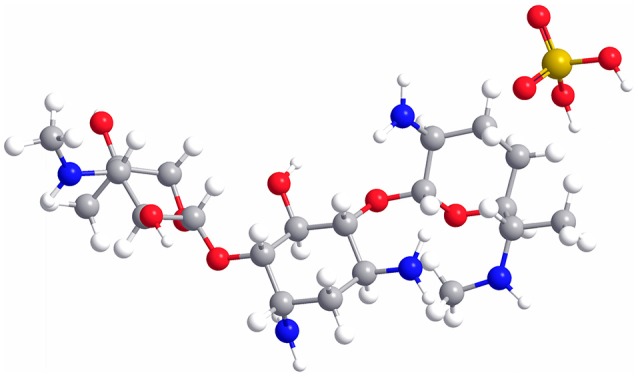
Molecular structure of GS. C, gray; H, white; O, red; N, blue; S, yellow.

As a water-soluble aminoglycoside antibiotic with potent broad-spectrum antibacterial activity (Rama Prasad et al., 2003; Gamazo et al., 2007), gentamicin is usually used in combination with sulfuric acid, forming gentamicin sulfate (GS, the molecular structure is shown in Figure 1), to treat the Gram-negative and Gram-positive bacterial infections (Drabu and Blakemore, 1990; Tang et al., 2014). However, gentamicin still has some shortcomings, such as low bioavailability, short half-life, and severe side effects (ototoxicity and nephrotoxicity), leading to its restricted application in clinical practice (Dahlgren et al., 1975; Tange et al., 1995; Dizaj et al., 2016). In addition, some bacteria are gentamicin-resistant due to its wide use in the treatment of human and animal diseases. Therefore, it remains a challenge to effectively improve the efficacy and minimize the adverse drug reaction of gentamicin.

Nanomaterials with special properties (Luo et al., 2006; Zhang et al., 2008) have been widely used in industrial, military, personal, medical, and antibacterial applications (Jiang et al., 2009; Ramaraju et al., 2015). Numerous studies have applied the nanoparticles (NPs) as the drug-carriers, showing promoted dissolution of drugs, enhanced absorption of drugs, increased drug targeted performance, controlled release capability of drugs and reduced side effects (Nakayama and Okano, 2005; Zhang et al., 2008; Liu et al., 2010). As an important inorganic mineral, calcium carbonate (CaCO_3_) mainly consists of three polymorphs, named calcite, aragonite, and vaterite (Dizaj et al., 2016), and only calcite is thermodynamically stable (Lauth et al., 2017). CaCO_3_ nanoparticles (CCNPs) are widely used in coating, plastic, paper, cosmetics, medicine, and food industries because of its characteristics of small size and high specific surface area (Fujiwara et al., 2010). Moreover, CCNPs can be also used as the delivery carriers of drugs, hydrophobic organic compounds and proteins due to its low toxicity and slow biodegrability (Shen et al., 2007; Qian et al., 2011; Dizaj et al., 2015; Palmqvist et al., 2017).

Previous studies have incorporated two antibiotics (metronidazole and gentamicin) into the non-sintered porous synthetic carbonate as the drug-carrier without alteration or degradation (Lucas et al., 2001). In addition, the GS-loaded CCNPs (CGPs) are used as efficient delivery system against the Gram-positive bacterium (*Staphylococcus aureus*), and the physicochemical properties and antimicrobial effect have been evaluated (Dizaj et al., 2016). However, the antibacterial mechanism remains unclear. In the present study, we also incorporated GS into CCNPs and explored the antimicrobial and synergistic mechanism. CCNPs were synthesized by carbonization method, and Gram-positive bacterium (*Bacillus subtillis*) was selected as the target bacterium. Our data provided valuable insights into the real application of CaCO_3_ as drug carrier.

## Materials and methods

### Materials and chemicals

GS was supplied from J&K Co. (Beijing, China). Calcium hydroxide [Ca(OH)_2_], ethylene diamine tetraacetic acid (EDTA) and other reagents were purchased from Sinopharm Chemical Reagent Co., Ltd. All the chemicals were of reagent grade.

### Preparation and characterization of CCNPs and CGPs

CCNPs were synthesized by carbonization method. Previous studies have shown that the addition of EDTA can significantly accelerate the carbonation rate, enhance the nucleation of CaCO_3_ particles and reduce the reaction time (Xiang et al., 2002; Feng et al., 2007; El-Sheikh et al., 2013; Zhou et al., 2014). Therefore, we selected EDTA as the suitable chemical additive to prepare the CCNPs according to the previous reports.

Briefly, 6.5% Ca(OH)_2_ was mixed with 1% EDTA in a beaker through continuously stirring. The beaker was then placed into a sealed vessel, which was connected to a CO_2_ high-pressure bomb. The CO_2_ ventilation was adjusted to 40 mL/min, and the pH was monitored until the pH-value reached 7.5. After carbonation treatment, the sediment was separated by centrifugation and rinsed with distilled water for three times.

The morphology of synthesized CCNPs was characterized on a Hitachi S-4800 scanning electron microscope (SEM) and a JEM-2010 transmission electron microscope (TEM). Meanwhile, the synthesized CCNPs were identified by X-ray powder diffraction (XRD) analysis, and the patterns of samples were recorded using a PANalytical X'Pert PRO diffractometer with Cu-Kα radiation (40 kV, 40 mA) in a continuous scanning mode. The 2θ scanning ranged from 5 to 85° in steps of 0.008° with a collection time of 50 s per step. The average size of NPs was calculated from the peak broadening using the Scherrer equation:

(1) $$\begin{array}{r} d=\frac{0.89\times \lambda }{B\times \text{cos}\theta } \end{array}$$

where *d* is the crystallite size; λ is the X-ray wavelength; Cu Kα λ = 0.15418 nm; *B* is the width of the line at the half-maximum intensity (in radians); and θ is the half of the diffraction angle (Arda et al., 2013; Moosavi et al., 2017).

Additionally, GS was incorporated into CCNPs by direct mixing. Briefly, 0.1 g CCNPs was added into 100 mL distilled water and stirred at 30°C. Subsequently, 0.1, 0.05, or 0.067 g GS (the mass ratio of GS: CCNPs = 1:1, 1:2, and 2:3) was added to the solution, and the mixture was mildly stirred at 300 rpm. The resultant CGPs were obtained by centrifugation at 12,000 rpm for 5 min.

Moreover, the prepared CCNPs, GS, and CGPs were analyzed by Fourier-transform infrared spectroscopy (FT-IR). Briefly, the samples were prepared in a KBr pellet with a sample/KBr ratio of 2:100, a thin pellet was placed in the IR beam on FT-IR spectrometer (Perkin-Elmer Spectrum One, USA) within a range of 4,000–500 cm^−1^ at a spectral resolution of 4 cm^−1^ (number of scans, 45), and the baseline was corrected.

### The standard curve of GS

**Figure 2:**
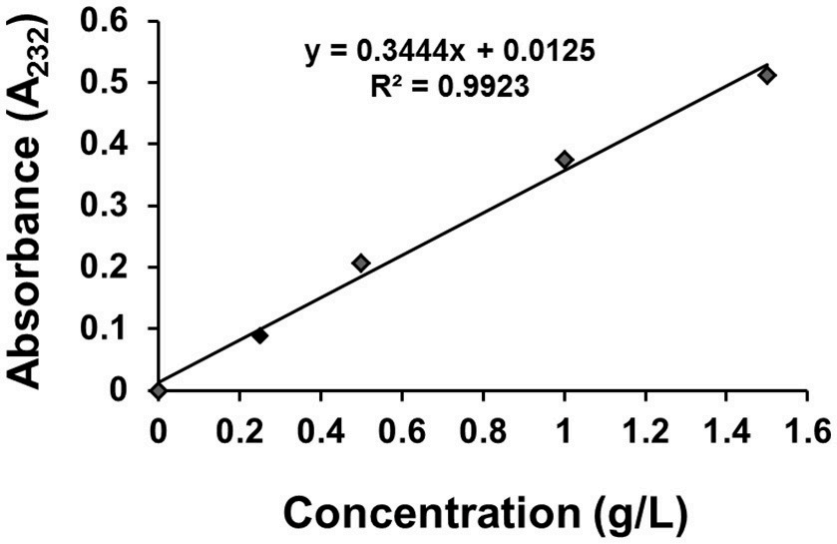
The standard curve of GS.

The standard curve of GS dispersion in distilled water was examined by using UV/VIS spectrophotometer (Perkin-Elmer Lamda 35). The concentration of GS ranged from 0.25 to 2.0 g/L, and the sample was completely dissolved using magnetic stirrer. Then 1 mL solution was transferred into a 50-mL volumetric flask, 0.5 mL H_2_O was then added to the flask, and the mixture was placed in water bath at 37°C for 10 min and finally diluted with water to volume. The GS had the maximum absorbance at 232 nm, and the standard curve was shown in Figure 2.

### Drug loading, entrapment efficiency, and GS release

Briefly, 1 mL of sulfuric acid was added into CGPs, the CGPs was completely dissolved after incubation in water bath for 10 min, and then the amount of loaded GS was detected by a UV/VIS spectrophotometer at 232 nm and quantified from standard curve.

The percentages of drug loading and entrapment efficiency were calculated according to the following equations:

(2) $$\begin{array}{r} \text{Drug loading}=m_{1}/m_{2}\times 100\% \end{array}$$

(3) $$\begin{array}{r} \text{Entrapment efficiency}=m_{1}/m_{0}\times 100\% \end{array}$$

Where *m*_0_ is the actual mass of the added GS; *m*_1_ is the mass of the GS on CGPs; and *m*_2_ is the mass of CCNP carrier.

### Bacterial culture conditions and bacterial inhibition study

The *B. subtillis* used in whole experiment was stocked in our laboratory. The liquid Luria–Bertani medium (LB, pH 7.0) consisted of 10 g/L NaCl, 10 g/L tryptone, and 5 g/L yeast extract, and the solid LB medium contained extra 20 g/L agar powder in addition to liquid LB medium. Subsequently, 1% *B. subtillis* in glycerine was inoculated into liquid LB medium at 37°C. After shaking at 180 rpm for 24 h, bacterial suspension was centrifuged at 6,000 rpm for 5 min and then washed with Tris-HCl twice to remove excess reactants.

The antibacterial activity of CCNPs, GS, and CGPs was determined at a GS concentration of 0.1 g/L. GS, CCNPs, and GCPs were added to the solution containing the same volume of bacteria. Double distilled water (ddH_2_O) was prepared as control, and bacteria-absent groups with the same other parameters were used as background in order to remove turbidity from CCNPs and GS. The mixture was incubated at 37°C on a shaker at 160 rpm. The OD-value at a wavelength of 600 nm was recorded by using UV/VIS spectrophotometer in order to detect the bacterial turbidity after 24-h incubation, and the inhibition rate (η) was calculated as follows:

$$\begin{array}{l} \eta =\left({\text{A}}_{0}-{\text{A}}_{1}\right)/{\text{A}}_{0}\times 100\% \end{array}$$

where A_0_ refers to the OD_600_ of bacteria in the absence of GS or CCNPs; and A_1_ refers to the OD_600_ of bacteria incubated with GS, CCNPs, or CGPs.

Additionally, LB-agar plates were used to further confirm antibacterial activity before and after 24-h incubation. Then 100 μL of bacterial suspension with appropriate dilution was spread on sterilized plates with LB-agar medium and incubated at 37°C overnight. Bacterial colonies grown in each plate were observed and images were taken.

### Zeta-potential (ZP) analysis

The changes of surface charge of CCNPs, GS and CGPs after the exposure to bacteria were determined by a ZetaPlus Zeta Potential Analyzer produced by Malvern Instruments Corporation, and the incubation time was 24 h. For each sample, an appropriate amount of undiluted solution was placed into the cuvette, and an average ZP-value was obtained from three individual measurements. Water was used as solution medium for all ZP measurements.

### Microscopic investigation of bacteria

The bacteria were imaged by Hitachi model H-7650 TEM. After exposure to CCNPs, GS and CGPs for 12 h, bacteria were fixed with 2.5% glutaraldehydein phosphate buffer (0.1 M, pH 7.0) for 24 h and washed in the phosphate buffer (0.1 M, pH 7.0) for three times (15 min for each). Then samples were post-fixed with 1% OsO_4_ in phosphate buffer for 2 h, and then fixed samples were respectively dehydrated in 30, 50, 70, 80, 95, and 100% acetone for 3 min. Then cells were embedded in Spurr resin. The specimen was sectioned by LEICA EM UC7 ultratome, and sections were stained by uranyl acetate and alkaline lead citrate for 5 and 10 min, respectively. Finally, the prepared sections were observed with TEM.

Moreover, the confocal laser scanning microscopy was also applied to observe the bacteria. LIVE/DEAD Bac Light Bacterial Viability Kit (L7012) was used in our experiment. After exposure to CCNPs, GS, and CGPs for 12 h, the bacteria were collected and incubated with 10 μL Syto 9 at 37°C for 10 min (λ ex 485 nm, λ em 498 nm, and the final concentration was 5 μg/mL). Subsequently, the bacterial suspension was centrifuged at 5,000 g for 5 min at 4°C, and the supernatant was discarded. Then the samples were resuspended in 1.0 mL PBS and incubated with 10 μL propidium iodide (PI) for 10 min in the dark (λ ex 535 nm, λ em 617 nm, and the final concentration of PI was 10 μg/mL). The unincorporated dyes were removed by washing with PBS. One droplet of cell suspension (5 μL) was dropped on the freshly treated glass slide, and then it was covered with coverslip. Finally, the glass piece was sealed using the petroxolin. The samples were recorded using a confocal laser scanning microscope (Leica SP8).

### Statistical analysis

All data were expressed as mean ± standard deviation (SD). Data were arranged with Microsoft Excel 2010 and analyzed with SPSS 18.0 by one-way ANOVA. A *p*-value of < 0.05 was considered as statistically significant.

## Results and discussion

### Characterization of MSNs

**Figure 3:**
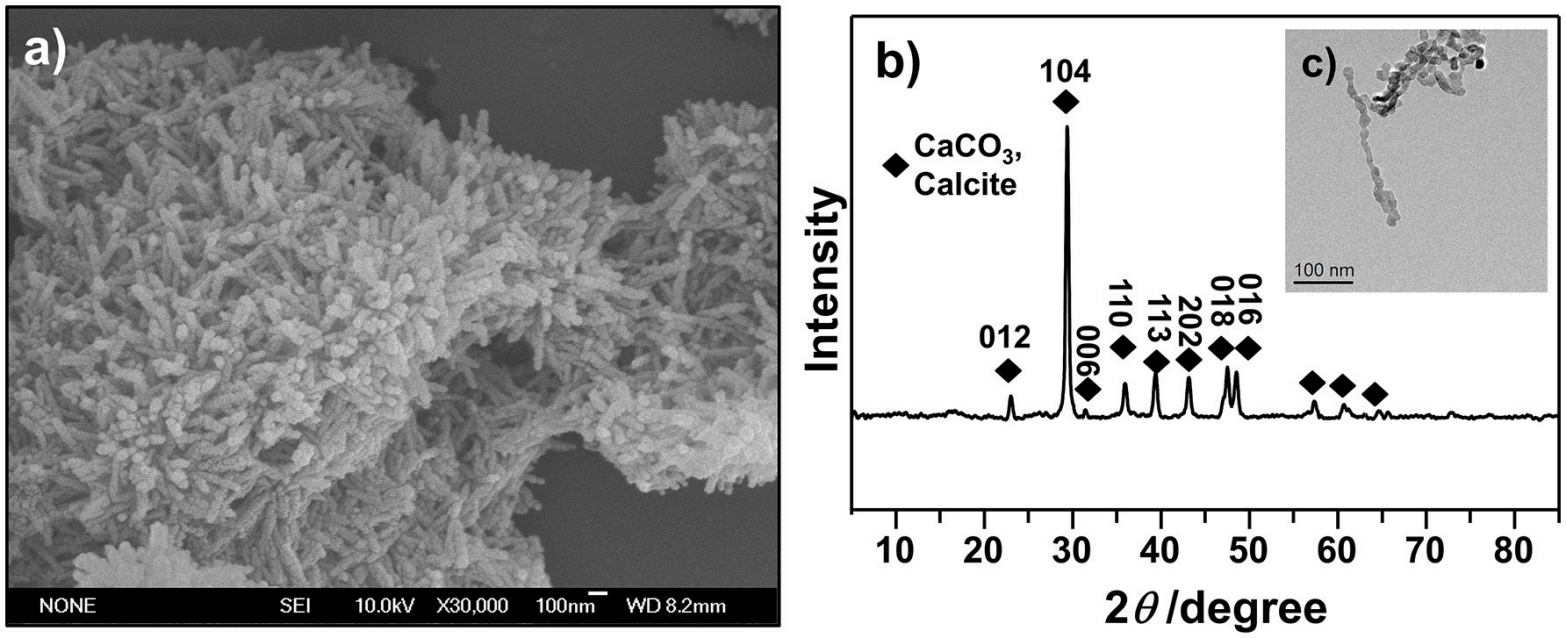
SEM **(a)**, XRD **(b)**, and TEM **(c)** images were used to illustrate the morphology and confirm the crystalline structures of as-synthesized CCNPs.

Rhombohedron is the dominant morphology of calcite polymorph, and truncated prismatic, scalenohedral, spherical, or chain-like agglomerates also exist in calcite (Ukrainczyk et al., 2009; Dizaj et al., 2016). We investigated the morphology of synthesized CCNPs by SEM and TEM and found that the synthesized particles exhibited chain-like structure of agglomerates with good dispersion (Figures 3a,c). Moreover, the diffraction pattern of XRD could be well indexed to calcite crystals. Meanwhile, the size of the crystallites was calculated through the application of the Scherrer's equation to the diffraction peak corresponding to the (104) plane, and its size was estimated to be about 62.5 nm (Figure 3b), confirming CaCO_3_ as a nanometer-level particle. Such structure could help particles disperse GS more evenly.

### FT-IR analysis

**Figure 4:**
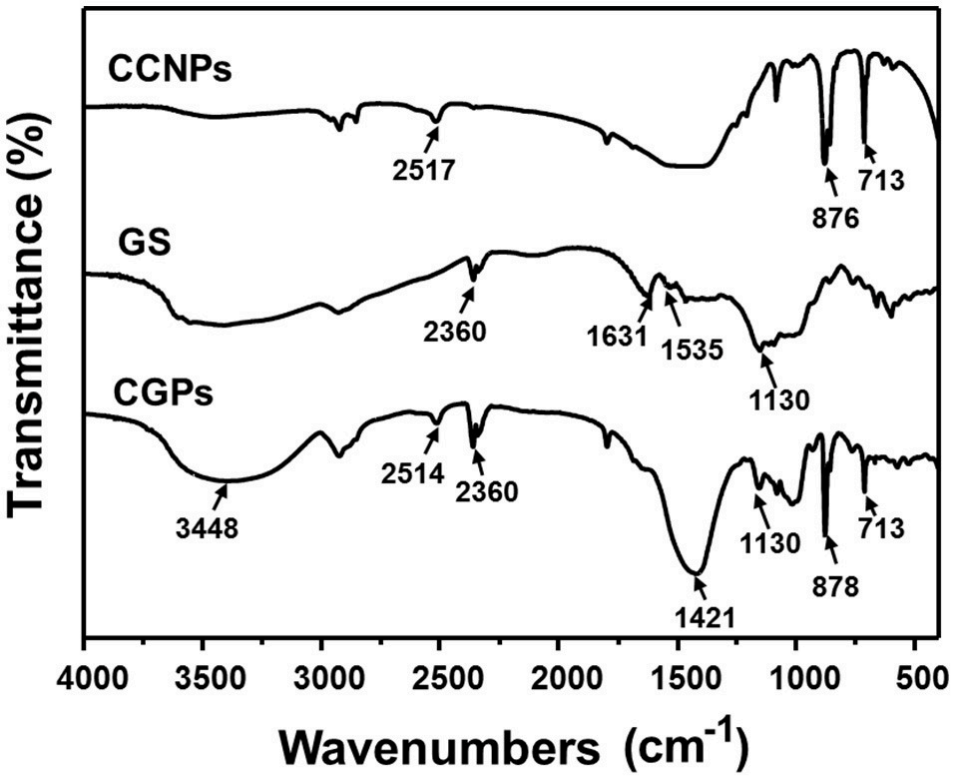
FT-IR spectra of CCNPs, GS, and CGPs.

The possible interaction between GS and CCNPs was investigated by FT-IR spectroscopy. Figure 4 shows that the spectrum of CCNPs displayed the characteristic absorption bands at 875 and 713 cm^−1^ (Wang et al., 2010), and the bands at 876 cm^−1^ have been identified as the ${\text{CO}}_{3}^{2-}$ bending vibration of calcite polymorph (Chen and Xiang, 2009). A broad band around 3,448 cm^−1^ confirmed the −OH stretching vibration of the absorbed water (Lin et al., 2014). In the spectrum of GS, it exhibited the bands at 1,631 and 1,535 cm^−1^ (N–H bending vibration bands) (Aquino et al., 2013; Dizaj et al., 2016), 1,130 cm^−1^ (C–O stretching vibration). The spectrum of CGPs showed most characteristic bands of GS and CCNPs, which indicated the effective loading of drug onto CCNPs. It was noticed that the bands at 1,631 and 1,535 cm^−1^ were gradually disappeared and a new band at 1,421 cm^−1^ was appeared, thus we speculated that hydrogen bonding interaction might exist between the drug and carrier during the preparation process.

### Drug loading and entrapment efficiency

In order to evaluate the usability of CCNPs as the nano-based carriers, we assessed the drug loading and entrapment efficiency in our drug delivery system. Table 1 shows that the loading efficiency of GS was gradually decreased with the increase of the CCNP dosage. For example, the loading efficiency of GS was decreased from 50.05 to 31.17% when the dosage of CCNPs was increased from 0.20 to 0.40 mg. However, the total loading capacity and entrapment efficiency of GS were gradually improved, and the entrapment efficiency of GS could reach 62.34% when the dosage of CCNPs was 0.40 mg. Dizaj et al. have reported a similar system for the loading of GS onto CCNPs, with the drug loading and entrapment efficiency of 25.3 and 38.6% (Dizaj et al., 2016), respectively. Our results indicated that the synthesized CCNPs provided high drug loading and entrapment efficiency toward GS.

**Table 1:** The drug loading and entrapment efficiency of GS are affected by different dosages of CaCO_3_.

**CCNPs/mg**	**GS loading capacity/mg**	**GS loading efficiency/%**	**GS entrapment efficiency/%**
0.20	0.1001	50.05 ± 1.85^a^	50.05 ± 1.65^c^
0.24	0.1045	43.55 ± 1.96^b^	52.26 ± 0.85^c^
0.28	0.1075	38.40 ± 2.83^c^	53.76 ± 2.20^bc^
0.36	0.1125	31.25 ± 1.69^d^	56.25 ± 2.14^b^
0.40	0.1247	31.17 ± 1.66^d^	62.34 ± 1.00^a^

*Different letters within column denote significant difference (p < 0.05)*.

### *In vitro* release and antibacterial activity of CGPs

**Figure 5:**
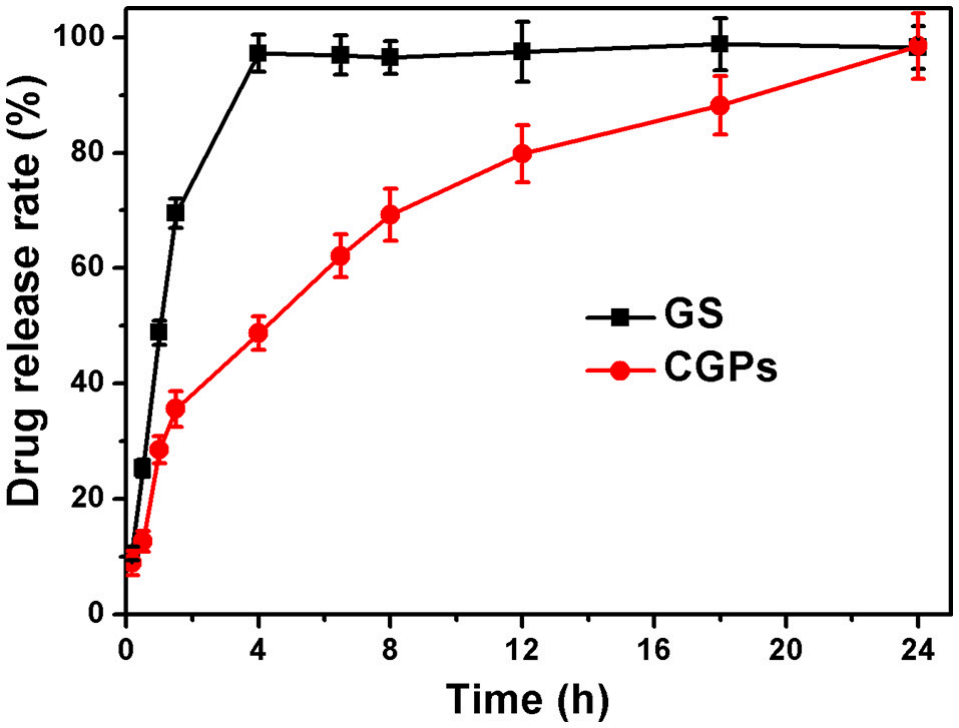
The release profiles with the same initial GS concentration. Data were expressed as mean ± standard deviation (*n* = 3).

The drug release was determined by the dissolution testing under the controlled conditions. Figure 5 illustrates the release patterns of pure GS and CGPs, showing that 50% GS was released from pure GS within 1 h, while 50% GS release from CGPs was found at 4 h. Meanwhile, the pure drug was completely released within 4 h, whereas the complete release of GS from CGPs was extended up to 24 h, suggesting that the GS release from CGPs was slower compared with pure GS. Previous studies have also exhibited that several drugs, such as granulocyte-colony stimulating factor, betamethasone phosphate, insulin, and validamycin, show sustained release after loading onto CCNPs (Haruta et al., 2003; Ueno et al., 2005; Qian et al., 2011), and the results in our study was also similar to a previous study (Dizaj et al., 2016).

**Figure 6:**
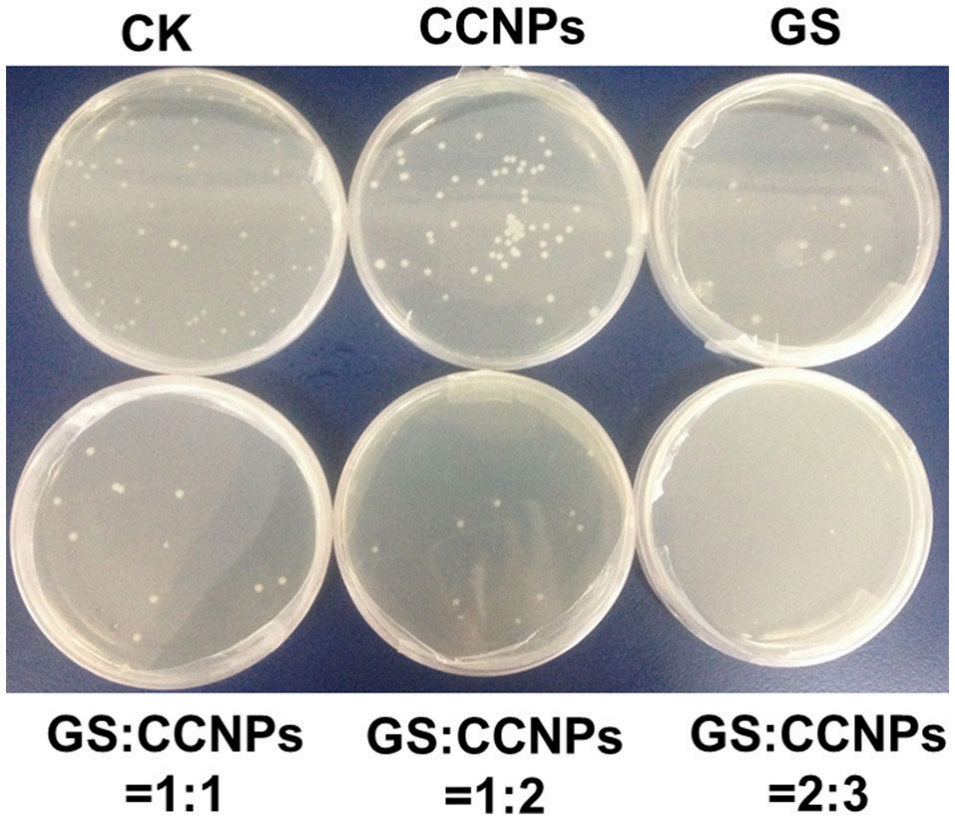
The colonies of *B. subtillis* treated by CCNPs, GS, and CGPs.

Moreover, we evaluated the antibacterial activity of GS, CCNPs, and CGPs in liquid medium or solid agar plate. Table 2 reveals that the antibacterial effect of CCNPs was not obvious (12.43%), whereas the GS had the slight antibacterial activity toward *B. subtillis* with an inhibition rate of 22.84%. However, GS significantly inhibited *B. subtillis* after loading onto CCNPs, showing an inhibition rate of 69.79%. This finding indicated that GS was successfully incorporated into the matrix of CCNPs, and such incorporation not only maintained its biological effect, but also increased the antibacterial activity to a great extent. Additionally, the solid agar plate experiment also confirmed that CGPs had a significant antibacterial efficiency (Figure 6), and the antibacterial activity toward *B. subtillis* could be ranked as follows: GS:CCNPs = 2:3>GS:CCNPs = 1:2 ≈GS:CCNPs = 1:1>GS>CCNPs. Taken together, CCNPs could increase the antibacterial activity of GS.

**Table 2:** The inhibitory effects of CCNPs, GS, and CGPs on *B. subtillis*.

**Samples**	**η/%**
CCNPs	12.43 ± 2.12
GS	22.84 ± 3.48
CGPs	69.79 ± 5.27

### Synergistic antibacterial mechanism

In order to explore the synergistic antibacterial mechanism of CCNPs, we further examined the ZPs of CCNPs, GS, and CGPs before and after exposure to bacteria. Table 3 shows that CCNPs, GS, and CGPs carried negative charges. After incubation with highly negatively charged *B. subtillis* for 12 h, all the three mixtures still carried negative charges. This result indicated that the binding of CCNPs, GS, and CGPs with bacteria could not be driven by the electrostatic interactions, but might by intermolecular forces.

**Table 3:** ZPs of CCNPs, GS, and CGPs before and after exposure to bacteria for 12 h.

**Samples**	**Zeta potentials**
Bacteria	−45.27 ± 0.41
CCNPs	−26.01 ± 1.06
CCNPs + Bacteria	−20.23 ± 1.02
GS	−3.3 ± 0.41
GS + Bacteria	−30.14 ± 2.64
CGPs	−12.74 ± 2.00
CGPs + Bacteria	−19.93 ± 0.69

**Figure 7:**
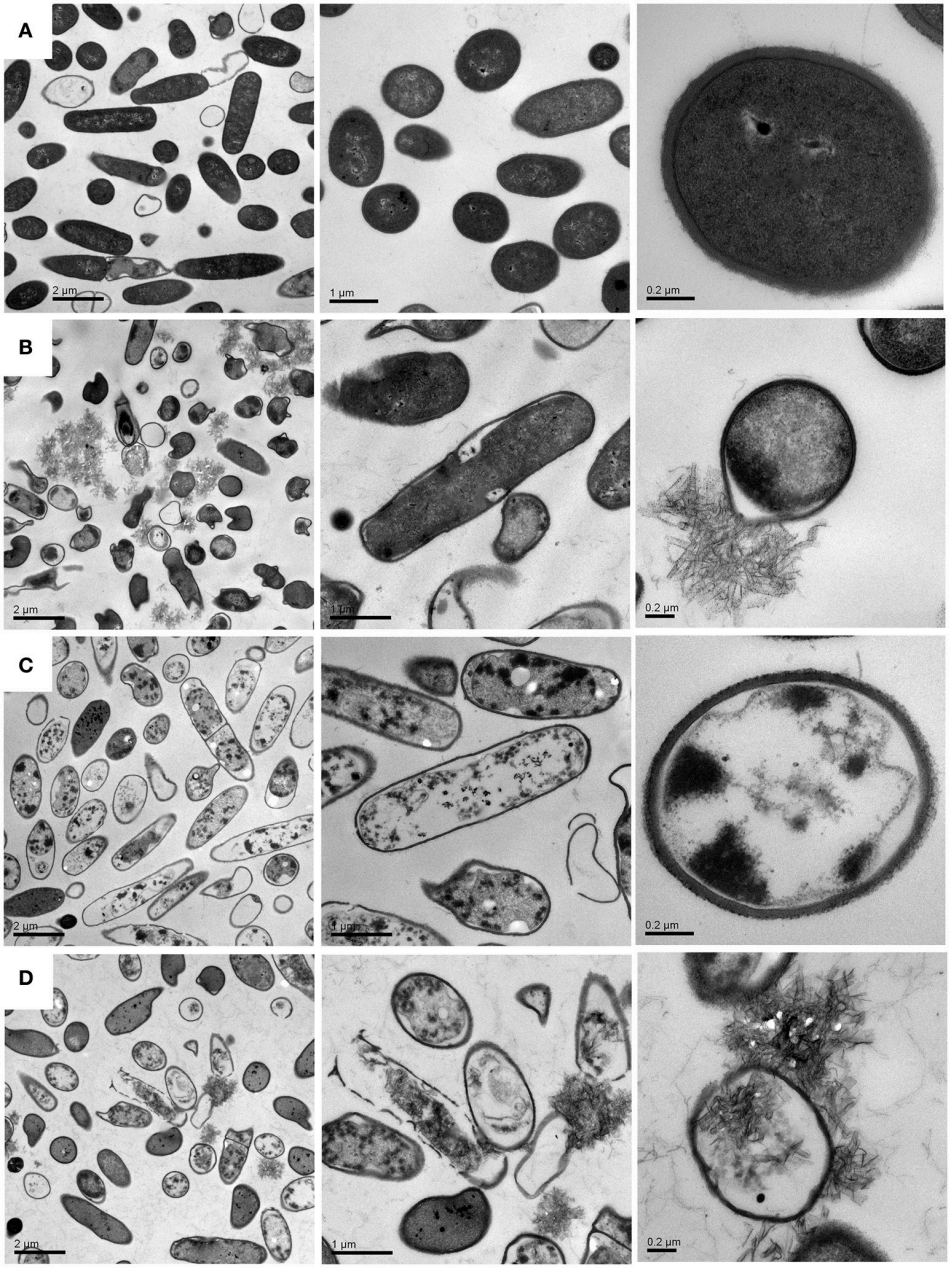
TEM images of *B. subtillis* before **(A)** and after treated with CCNPs **(B)**, GS **(C)**, and CGPs **(D)** for 12 h.

Although previous studies have also incorporated the drugs into CCNPs to develop the efficient delivery system, researchers focus on the physicochemical characterization and antimicrobial evaluation, while the microscopic investigations are rarely conducted. In the present study, we performed TEM and confocal microscopy in order to explore the detailed antibacterial mechanism. Figure 7A shows that bacteria in pure water without exposure to drugs or carriers exhibited relatively intact profile and clear cell walls. After interacting with CCNPs for 12 h (Figure 7B), the NPs with chain-like morphology were aggregated in the medium, and most of the bacteria still remained the intact profile. However, several cells were damaged, and cell wall remained relatively intact. For GS-treated bacteria (Figure 7C), analysis revealed that GS might be internalized into the cell, the cell wall still remained complete, and most of the cell inclusion leaked. However, when exposed to CGPs (Figure 7D), the damage level of the bacterial profile and cell wall was increased compared with other samples, and the chain-like agglomerates were also found inside the bacteria. This result indicated that CCNPs, GS, and CGPs had different damageability to bacteria, but the cell walls had no obvious disorganization, and CCNPs could increase the antibacterial activity of GS by enhancing the damage level of the bacterial profile.

**Figure 8:**
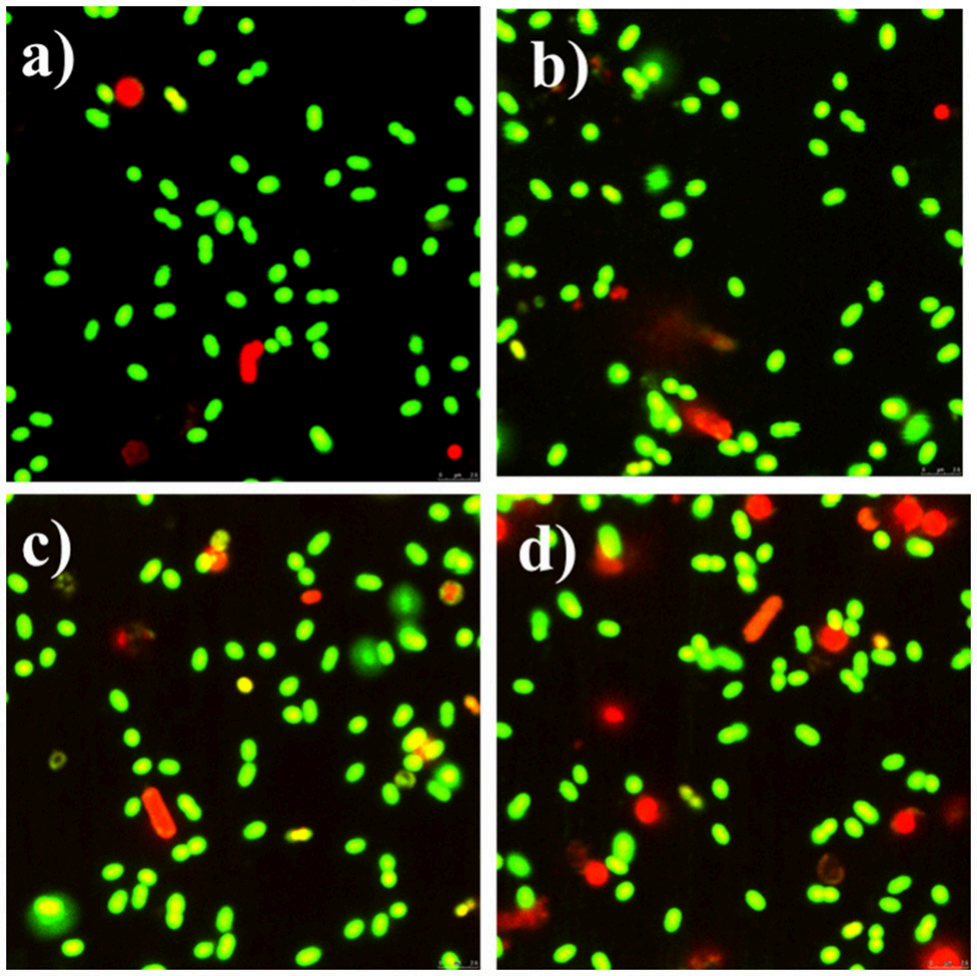
Fluorescence microscope of apoptotic and necrotic cells before **(a)** and after exposure to CCNPs **(b)**, GS **(c)**, and CGPs **(d)**. Briefly, 0.1 g/L sample was added to the bacterial suspension for 12 h. All stained samples were imaged at a comparable cell concentration.

Meanwhile, the bacteria were also examined by fluorescence microscope. Both Syto-9 and PI were used to reveal the changes of the viability and membrane integrity of bacterial cells. PI can only penetrate cells with disrupted membranes, showing intracellular staining of red fluorescence (George et al., 2010). In contrast, the green fluorescent staining of Syto-9 can enter live and dead bacterial cells. In brief, viable cells were stained green, while dead cells were stained red. Figure 8a shows that almost all of the control bacteria appeared strong green nuclei, indicating that PI could not penetrate the cell membranes of viable cells. CCNPs-treated bacteria (Figure 8b) also exhibited green fluorescence, and only a small portion of nuclei were stained red, suggesting that CCNPs had a weak destructive effect on the cell membranes, and majority of bacteria were viable. When exposed to GS (Figure 8c), the green fluorescence was greatly accumulated, while the yellow fluorescence was increased. The yellow fluorescence is generated when Syto-9 is not completely replaced by PI (Stiefel et al., 2015). Figure 8d reveals that the number of red nuclei was increased in CGP-treated bacteria, implying that some bacteria were in necrotic phase.

### The possible synergistic antibacterial mechanism of CGPs

**Figure 9:**
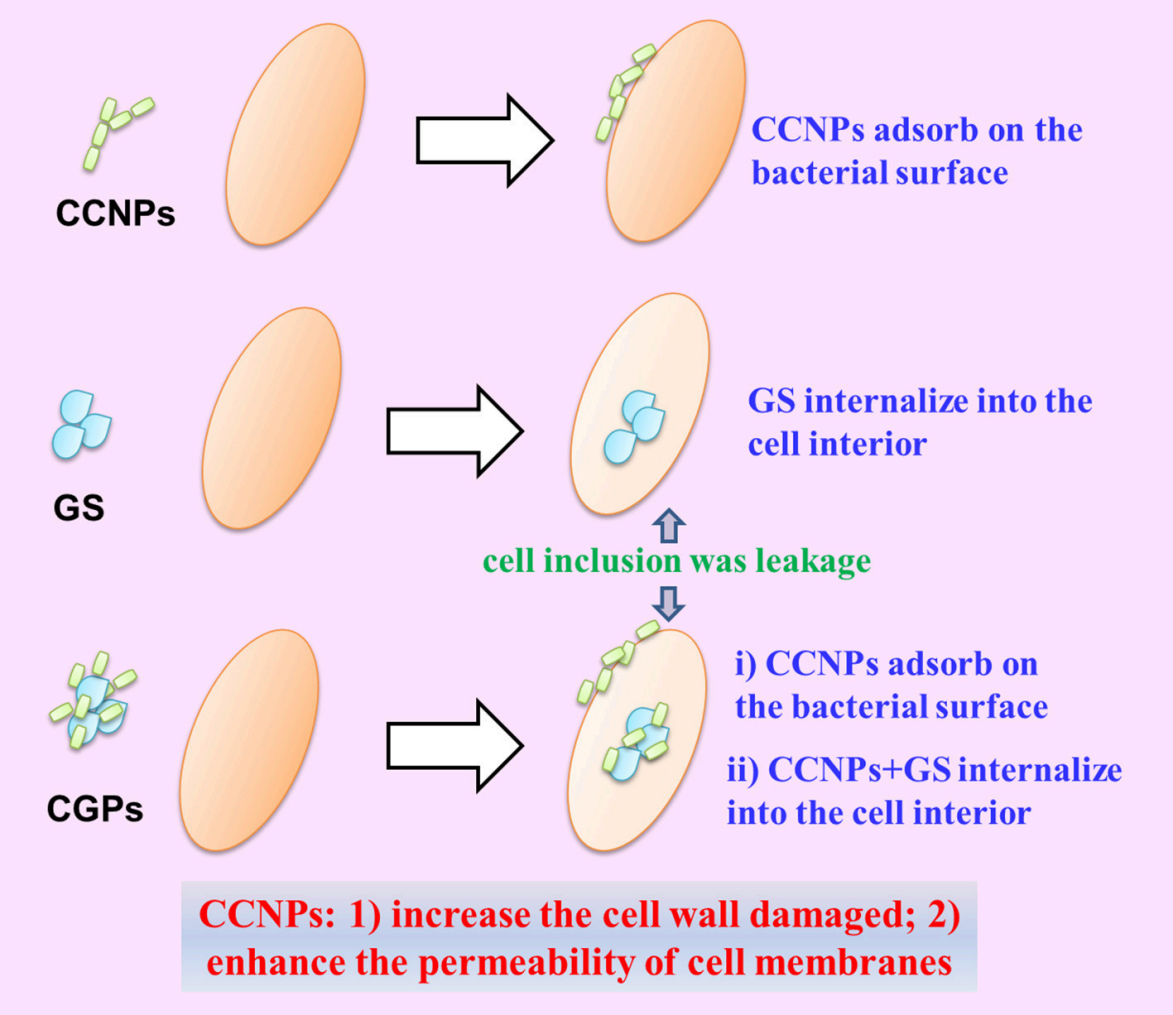
Schematic diagram of the synergistic antibacterial mechanism between NP aggregates and drug.

Based on the above-mentioned analyses, we proposed the possible synergistic antibacterial mechanism of CGPs (Figure 9). First, CCNPs with negative charge and small size might be adsorbed onto bacterial surfaces via intermolecular forces. Therefore, CGPs were more likely to interact with the bacterial surfaces. Second, CCNP carriers would increase the cell wall damage and enhance the permeability of cell membranes by GS, leading to the death of bacteria.

## Conclusions

It is critical to develop an effective targeted delivery of antibiotics in recent years. In the present study, CCNPs were used as carriers to incorporate GS, and physicochemical characterization and antibacterial effect of CGPs were evaluated. Our results indicated that CCNPs could prolong the release of GS and increase the antibacterial activity of GS. Moreover, the synergistic antibacterial mechanism of CGPs was proposed through microscopic investigations. Taken together, CCNPs could be used as potential carriers in drug delivery system, and our findings offered valuable insights into the real application of nano-CaCO_3_.

## Author contributions

XP designed the study, analyzed the data, and wrote the text. SC participated in the manuscript preparation. DL, WR, YZ, ZY, and LL performed the related experiments, and the sequential arrangement reflects their workload. XG and ZC participated in the manuscript preparation. All authors contributed to its final form and gave final approval for its publication.

### Conflict of interest statement

The authors declare that the research was conducted in the absence of any commercial or financial relationships that could be construed as a potential conflict of interest.
